# Stereotactic Body Radiotherapy for Renal Cell Carcinoma—A Review of Use in the Primary, Cytoreductive and Oligometastatic Settings

**DOI:** 10.3390/cancers16193334

**Published:** 2024-09-29

**Authors:** Conrad Josef Q. Villafuerte, Anand Swaminath

**Affiliations:** Department of Oncology, Division of Radiation Oncology, Juravinski Cancer Centre, McMaster University, Hamilton, ON L8V 5C2, Canada

**Keywords:** kidney cancer, renal cancer, renal carcinoma, renal cell carcinoma, stereotactic radiotherapy, stereotactic body radiotherapy, stereotactic ablative radiotherapy, oligometastases, oligoprogression, cytoreduction

## Abstract

**Simple Summary:**

Stereotactic body radiotherapy (SBRT) is a treatment technique that delivers higher doses of conformal radiation precisely to specific targets. SBRT is utilized for the treatment of the more common cancers such as lung, liver and prostate malignancies, both in the early stage and the oligometastatic setting. The aim of this review is to highlight the current role of SBRT for renal cell carcinoma (RCC), a disease which was previously thought to be radioresistant, particularly to conventionally fractionated radiotherapy. There is now an increasing body of literature encompassing published phase II trials, reviews and meta-analyses demonstrating the effectiveness of SBRT in both early-stage RCC (as an alternative to patients who are not surgical candidates) and within the context of metastasis-directed therapy (MDT) for patients with oligometastatic and oligoprogressive RCC. Ongoing randomized trials are also exploring the role of SBRT as a cytoreductive treatment in metastatic RCC.

**Abstract:**

Renal cell carcinoma (RCC) has been increasing in incidence by around 1.5% per year for several years. However, the mortality rate has been decreasing by 1.6% per year, and this can be attributed to stage migration and improvements in treatment. One treatment modality that has emerged in recent years is stereotactic body radiotherapy (SBRT), which is an advanced radiotherapy technique that allows the delivery of high-dose radiation to the tumor while minimizing doses to the organs at risk. SBRT has developed a role in the treatment of early-stage, oligometastatic and oligoprogressive RCC. In localized disease, phase II trials and meta-analyses have shown that SBRT provides a very high probability of long-term local control with a low risk of severe late toxicity. In oligometastatic (OMD) RCC, the same level of evidence has similarly shown good local control and minimal toxicity. SBRT could also delay the necessity to start or switch systemic treatments. Medical societies have started to incorporate SBRT in their guidelines in the treatment of localized disease and OMD. A possible future role of SBRT involves cytoreduction. It is theorized that SBRT can lower tumor burden and enhance immune-related response, but it cannot be recommended until the results of the phase II trials are published.

## 1. Introduction

### Renal Cell Carcinoma Statistics and Diagnosis

Renal cell carcinoma (RCC) is the predominant type of kidney cancer [1]. It ranks as the 14th most common malignancy worldwide, with a 5-year prevalence of 1,369,974 in 2022. Incidence has been increasing for decades, with 1.5% more localized tumors detected per year (2015 to 2019). This is partly due to the increased performance of medical imaging, which identifies more asymptomatic renal masses [1,2,3,4].

If not detected through imaging, RCC can present as hematuria, anemia, pain or discomfort, a lower back or abdominal mass, tiredness or fatigue, weight loss and fever [1,3]. Confirmation of malignancy is performed through a biopsy but other investigations are also needed, such as imaging (at least of the abdomen and chest) and serum and urine tests [4].

In terms of mortality, kidney cancer ranks 16th in the world among malignancies with 155,953 deaths in 2022 [2]. However, the mortality rate has been declining since the mid-1990s by around 1.6% [1]. This improvement may be attributed to stage migration and better treatments for advanced disease [3,5]. The current 5-year survival rate is 79% [1].

The general approach to the management of RCC is different between patients with localized disease and those with metastatic disease [1]. Surgery is the mainstay for localized disease while systemic treatment is the primary strategy for metastatic disease [1,3,4].

One treatment modality that has been accruing positive evidence and support of use in the primary and metastatic settings is stereotactic body radiotherapy (SBRT). It is a radiotherapeutic technique that utilizes high-dose ionizing radiation to provide ablative treatments to cancer targets. The main drivers of SBRT use in recent years are technological advancements in radiotherapy planning and delivery that makes it possible to precisely deliver the high doses of radiation at a particular target volume. In so doing, the normal organs are spared from the high-dose region and thus the risk of severe toxicity is limited [6,7,8,9].

In this review, we will discuss the evidence, management parameters and current role and place of SBRT in the management of both localized and metastatic RCC.

## 2. Renal Cell Carcinoma Treatment Overview

### 2.1. Staging and Prognostication

Staging for RCC is conducted using the American Joint Committee on Cancer TNM staging, which incorporates the primary tumor extent, regional node involvement and distant metastases [10].

For the prognostication of metastatic cases, the International Metastatic RCC Database Consortium (IMDC) model can be followed. Scoring considers four laboratory factors (hemoglobin, neutrophil, platelets and calcium) and two clinical factors (period from initial RCC diagnosis and metastasis development, and Karnofsky performance status [KPS]). Patients are then classified into three groups: favorable (0 factors), intermediate risk (1–2 factors) and poor risk (≥3 factors) [11,12]. The IMDC model is more commonly used compared to the Memorial Sloan Kettering Cancer Center (MSKCC) model [13].

### 2.2. Current Approaches for Localized Renal Cell Carcinoma

For patients with early stage disease, surgery either via partial or radical nephrectomy is the mainstay of management. Partial nephrectomy offers the prospect of conserving renal function and is an option for those with T1/T2 disease and select T3 cases [4,14,15,16].

Some patients, however, may not be able to undergo surgery due to advanced age or other comorbidities including cardiac, respiratory or chronic renal disease. This is particularly notable because RCC has the highest probability of developing between 65 and 84 years of age [1,17]. In medically inoperable patients with small tumors (typically 4 cm or less), alternative treatment options include thermal ablation techniques such as radiofrequency ablation, cryoablation, or microwave ablation [18]. The ability to perform thermal ablation can be limited by tumor location, which should be away from the hilum or ureter, and the potential for morbidity in high-risk patients because of the requirement for laparoscopic or percutaneous access [17,19]. Active surveillance is also an option for small renal masses, but concerns exist regarding higher malignant potential when tumor size exceeds 3–4 cm or with higher growth rates (i.e., 0.5–1 cm/year) [20]. Biopsy is advised if non-surgical options are chosen [19].

Targeted therapy or immunotherapy, particularly Pembrolizumab [21], may be advised as adjuvant treatments if high-risk factors are present, such as T4 or high-grade disease, nodal involvement, and sarcomatoid features [4,14,22]. Neoadjuvant systemic treatment is also currently being investigated further after the success of small trials [23,24,25].

### 2.3. Current Approaches for Metastatic Renal Cell Carcinoma

Around 20–30% of patients with RCC present with de novo metastatic disease [26,27] and after definitive surgery for localized disease, an additional 12–30% may develop metastasis [28,29,30]. The most common sites of metastases are the lungs, lymph nodes and bone [27,31]. The presentation of recurrence can be affected by the type of surgery for initially localized disease. As an example, more local recurrences and more metastases outside the abdominal and thoracic cavities are observed after minimally invasive surgery, which is uncommon [32]. The overall prognosis of patients with metastatic disease is poor, with a 15% 5-year survival [33,34].

The current first-line treatment for metastatic disease is systemic therapy with immunotherapy and targeted therapy. Through the years, the primary option has shifted from interferon or interleukin-2 in the mid-2000s to vascular endothelial growth factor (VEGF) receptors in the late 2010s, and currently immune checkpoint inhibitors (ICIs) [4,14,35,36,37,38,39,40]. In select cases who are asymptomatic, with limited tumor burden and slow-growing and favorable pathology, active surveillance may be an option [19,41,42,43].

## 3. History of Radiotherapy Use in Renal Cell Carcinoma

### 3.1. Radioresistance of RCC with Conventional Fractionation and the Initial In Vitro Studies with Hypofractionation

Radiation therapy was initially thought to be an ineffective treatment for RCC. Contributory to this belief were both laboratory studies which showed RCC to be inherently resistant to conventional fractionation and clinical trials which did not show any benefit. An in vitro paper, for example, revealed that RCC had a mean sensitivity of 4 Gy, making it among the most radioresistant of the tumor cell lines examined [44]. Several adjuvant radiotherapy (RT) clinical trials in the 1970–1980s and a subsequent meta-analysis revealed an overall reduction in locoregional recurrences but no disease-free survival (DFS) or overall survival (OS) benefit. A few trials even showed survival detriment with undesirable toxicity [45,46,47].

As such, external-beam RT did not have a large role in the treatment of RCC except for the palliation of symptoms [48]. This began to change with the publication of pre-clinical studies showing effectiveness on RCC cell lines using a higher dose per fraction treatment.

An investigation on mice injected with human RCC cells compared a population that was observed vs. those treated with 48 Gy/3 fractions (fx). The findings revealed both cytological changes in the cells and consistent mass shrinkage [49]. A similar Japanese study on nude mice showed survival curve responses if a >5 Gy dose per fraction was used, and a dose–response relationship with higher doses [50]. A radioimmunotherapy paper also revealed the efficacy of higher fractionation in killing human RCC cell xenografts [51]. Potential explanations as to why RCC responds to higher fractionation may involve speedy microvascular cell apoptosis or death secondary to membrane ceramide accumulation and more acid sphingomyelinase activity [6,7,52,53].

These studies heralded subsequent clinical research into the utilization of hypofractionated regimens and SBRT in RCC [54,55].

### 3.2. Stereotactic Body Radiotherapy: Definition and Technology

SBRT or stereotactic ablative radiotherapy (SABR) is currently defined as an external-beam radiotherapy technique that can accurately deliver high doses of radiation to an extracranial target, such as the kidney, in one to five fractions.

The term “stereotactic” relates to a treatment procedure wherein a lesion is identified and localized through a three-dimensional reference system. In SBRT, the term still applies despite not having external stereotactic coordinates, since these are replaced by internal body coordinates through image guidance. Historically, stereotactic treatments were first developed for the management of brain or spine lesions and were called stereotactic radiosurgery (SRS). It was only later in the 1990s that the principles and practice was applied to extracranial structures [56,57].

Crucial requirements for optimal SBRT include (1) relatively smaller target volumes compared to those treated with conventional fractionation; (2) well-delineated targets through the use of multi-modality imaging at the time of radiation planning; (3) accurate treatment delivery with daily image guidance on the treatment unit; (4) organs at risk (OARs) omitted from the target (gastrointestinal luminal organs and kidney parenchyma are of particular importance for kidney SBRT); and (5) highly conformal therapy through the combination of using advanced dose planning software and treatment delivery platforms [8,9,17].

These requirements are needed to ensure that the target receives higher radiation doses that would increase the chance of tumor control, while also minimizing the dose received by the OARs, thereby reducing the probability of adverse events.

## 4. SBRT in Early-Stage Renal Cell Carcinoma

SBRT has been carving out a niche as a reasonable treatment alternative for patients with early-stage RCC who are medically inoperable.

The other options besides SBRT for inoperable patients are interventional ablative techniques and active surveillance with intent to treat on progression. An in-depth discussion of these two options is beyond the scope of this review, but a summary of the possible patient selection factors, limitations and nuances are shown in Table 1. Table 2 illustrates the general considerations for SBRT. It is also important to state that high-quality evidence comparing the effectiveness of one intervention over another is still lacking.

### 4.1. Phase I-II Trials Supporting SBRT for Primary RCC

The initial SBRT phase I dose-escalation studies were mostly started in the 2000s. In general, results were favorable, showing good local control (LC) and no dose-limiting toxicity (DLT) even with higher total doses. One dose-escalation paper included patients with Karnofsky performance status (KPS) ≥70 with ≤5 cm tumors that were radiographically concerning or biopsy-proven to be RCCs. Doses reached as high as 48 Gy/3 fx. Only two local failures in 15 patients were observed with no DLTs [83,84]. A similar paper had doses reaching 60 Gy/3 fx with a local control (LC) of 90% at 3 years. Only one grade 4 toxicity (duodenal ulcer) was observed [81,85]. The Trans-Tasman FASTRACK trial utilized a 26 Gy/1 fraction for tumors <5 cm and 42 Gy/3 if ≥5 cm. Thirty-three patients and thirty-four RCCs were treated. At the 2-year median follow-up (f/u), freedom from local progression and distant progression was 100% and 89%, respectively. OS was 92%. Only one grade 3 toxicity was seen with no grade 4–5 toxicity [86]. A more recent dose-escalation trial with 13 patients assessed four doses, with the highest at 48 Gy/4. At the 2-year f/u, no DLTs were observed, with the highest toxicities only reaching grade 2. There were 11 patients with stable disease and 2 with partial response [87].

Subsequent phase II and pilot randomized studies have been performed as well and they are shown in Table 3. A trial by Hannan et al. treated tumors ≤5 cm that were previously shown to increase in size on imaging and pathology-confirmed RCC. There were two dose options, 36 Gy/3 or 40 Gy/5. After a median f/u of 3 years, the LC in 1 year was 95%. There were no grade ≥2 adverse events (AEs) [74]. The continuation to the FASTRACK phase I study, FASTRACK II, was published recently with a median f/u of 43 months. It enrolled 70 patients with tumors ≤10 cm. The masses were biopsy-proven and not in contact with the bowel. Tumors ≤4 cm were treated with 26 Gy in one fraction while tumors >4 cm were treated with 42 Gy in three fractions. LC at 1 year was 100%. No grade ≥4 toxicity was seen and only seven (10%) patients had grade 3 toxicity [75]. A pilot randomized trial from Canada compared RFA vs. SBRT (25 Gy/1 fx) in 24 patients with masses ≤4 cm. The results showed both arms having no local failures and low toxicity rates at 1 year, including an acceptable mean reduction in eGFR. The full manuscript and a longer f/u are awaited as only the abstract has been published [80].

### 4.2. Meta-Analyses, Reviews and Guidelines

Several reviews and meta-analyses have been performed in order to summarize and harmonize the growing data and help guide treating clinicians and radiation oncologists [17,88]. A paper published in 2019 by Correa et al. found 384 RCC masses in 372 patients, with around 80% having local disease. Patient f/u was approximately 28 months. They found that the commonly used fractionations were 26 Gy/1 or 40 Gy/5. LC was estimated to be 97% with only a 1.5% chance of grade 3–4 AEs. The eGFR decline after SBRT was 7.7 mL/min. In patients who had a history of renal dysfunction, there was a 2.9% probability of dialysis [89].

The International Radiosurgery Consortium of the Kidney (IROCK) has published several analyses and consensus through the years [79]. The 2018 paper had 223 patients and the median f/u was 2.6 years. It was illustrated that the rates of LC, cancer-specific survival (CSS), and progression-free survival (PFS) were 97.8%, 95.7%, and 77.4% at 2 years and 97.8%, 91.9%, and 65.4% at 4 years. Larger tumors and use of multi-fraction SBRT was a factor for worse CSS and PFS. The eGFR decreased by a mean of 5.5 mL/min. Only 2.7% of patients underwent dialysis. The probability of grade 1–2 and 3–4 adverse toxicity was 35.6% and 1.3%, respectively [90]. The 2022 paper focused on patients who were followed for longer, with the median f/u at 5 years. Local failure was only 5.5% among 190 patients. Use of multi-fraction SBRT was a factor for more local failures, although those patients trended towards older age and poorer performance status. The eGFR decreased by a median of 14.2 mL/min, and only 4% had dialysis after SBRT. Only 1 patient (1%) had grade ≥3 toxicity [78]. Another IROCK study examined patients with solitary kidneys with 81 patients analyzed. The LC, PFS, CSS and OS at 2 years was 98%, 77.5%, 98.2% and 81.5%, respectively. The mean decline of eGFR was 5.8 mL/min. It was found that treatment of tumors >4 cm was associated with a worse decline of eGFR at ≥15 mL/min [77].

The data from the different trials and reviews have influenced a few medical societies to mention or incorporate SBRT in their clinical practice guidelines (CPGs). The European Society of Medical Oncology (ESMO) 2019 guideline describes RT as an alternative for patients with unresectable local or recurrent disease. This could be due to poor performance status or comorbidities, particularly if other local therapies like RFA are not appropriate [91]. The Canadian Urologic Association (CUA) considers SBRT to be one of the novel non-surgical therapies, but longer-term data are needed before further recommendations [59]. The European Association of Urology (EAU) 2022 guide broadly classified SBRT under minimally invasive ablative treatments as one of the substitute therapeutic approaches to surgery [19]. The National Comprehensive Cancer Network (NCCN) kidney cancer guide version 4.2024 specifies that SBRT can be considered for patients who are medically inoperable with stage I disease (category 2B) and those with stage II or III disease (category 3) [60].

SBRT-specific guidelines and recommendations have also been published of late. The International Society of Stereotactic Radiosurgery (ISRS) provided suggestions about dose, need for biopsy after SBRT, treatment of patients with solitary kidneys and f/u schedule [76]. Barbour et al. shared additional information involving SBRT simulation and planning, including dose constraints [17]. Some of these suggestions are highlighted within the considerations for SBRT illustrated in Table 2.

### 4.3. Safety and Toxicity

Based on various studies and papers, it can be summarized that the common acute toxicities of SBRT in the kidney mostly involve fatigue or nausea and vomiting. Uncommon acute side effects include dermatitis, abdominal discomfort/pain, and other gastrointestinal toxicity like diarrhea, and these tend to be mild in nature. The IROCK group quoted an overall 37% chance of grade 1–2 AEs. Correa et al. mention 37.5% and 8.8% chances of grade 1 or grade 2 toxicity, respectively. The probability of more severe grade 3–4 AEs is around 1.5% (range 0–25%). Examples include bowel damage leading to bleeding and perforation, rib fractures and spinal cord injury. In terms of renal function, the decline of renal function at around 2 years is −7.7 mL/min compared to −14.2 mL/min at 5 years. The need for dialysis is estimated to be 2.7–4% in 2–5 years; however, the attribution to SBRT is difficult to quantify given that many patients present with pre-existing moderate-to-severe chronic kidney disease [17,89,90,92].

To lower the chances of toxicity, it is suggested that cases are treated following the indications mentioned in the trials and guidelines (Table 2 and Table 3), namely, mass size <4–10 cm, lesions not touching or invading luminal gastrointestinal structures and a GFR of ≥30 mL/min. There should be more vigilance if one is considering SBRT in cases beyond the listed parameters. The treatment of larger tumors exposes more normal tissue to higher doses of radiation, such as normal kidney parenchyma, and thus may cause worse kidney function. Some authors have suggested that giving SBRT to patients with GFR <30 mL/min in SBRT may lead to more iatrogenic dialysis [17,75,76].

### 4.4. SBRT in Early-Stage Renal Cell Carcinoma Summary

SBRT for localized primary RCC is an evolving modality that has carved its place as a suitable alternative option for patients who are inoperable and within the criteria demonstrated in Table 3. The use of SBRT is already supported by several phase II trials and meta-analyses illustrating excellent LC and minimal adverse toxicity. Longer f/u and further phase III trials comparing SBRT and other local treatments are eagerly awaited to help further entrench SBRT among the treatment options and further solidify its role in localized RCC.

## 5. Cytoreductive SBRT in Metastatic Renal Cell Carcinoma

Cytoreductive nephrectomy (CN) was previously part of the standard treatment in newly diagnosed de novo RCC with synchronous metastases. A survival benefit was seen when combined with adjuvant interferon alph-2b in trials [93,94], and Pembrolizumab in retrospective data [95,96]. Practice changed when it was found that the tyrosine kinase inhibitor (TKI) Sunitinib, when given alone, was non-inferior to CN with Sunitinib [97,98]. Currently, only patients with good prognosis and whose metastatic burden can also be removed surgically (via metastasectomy) are suitable candidates to undergo CN [1,4,30,43].

Prospective studies on using SBRT similarly to CN are currently ongoing. Other than potentially reducing the number of active cancerous cells and addressing a possible source of further metastases, cytoreductive SBRT may also enhance the immune response, leading to an “abscopal effect”, described as targets and non-target lesions both responding and decreasing in size [99]. Changes in the immune system secondary to SBRT of the kidney have been observed in pilot studies and they may include modification and/or increases in immune-modulating cytokines such as calreticulin, tumor-associated antigens and proliferating T-cells, including the T-cell responses [100,101]. The abscopal effect and other SBRT immune-mediated changes may also lead to enhanced non-target response to immunotherapy.

There are two ongoing phase II randomized trials investigating cytoreductive SBRT of the kidney, primarily for patients diagnosed with de novo metastatic disease. The first trial is the CYTOSHRINK trial, which was started in Canada in 2019. It completed accrual in 2024 and the results are pending [102]. The second trial is the NRG-GU012/SAMURAI trial, which started in the middle of 2022. The estimated enrolment is 240 patients, it is approximately 10% accrued, and primary completion is expected by 2028 [103]. Table 4 shows the highlighted aspects of the two trials. The publication of these trials is highly awaited since cytoreductive SBRT is still largely considered to be investigational.

## 6. SBRT for Oligometastatic Renal Cell Carcinoma

### 6.1. Definition of Oligometastases

The oligometastatic state was originally expressed by Hellman and Weichselbaum in 1995. It was explained to be an intermediate state between local and systemic disease, where radical local treatment of the primary and all metastatic lesions might have a curative potential [104]. This definition has evolved since then, with the current ESTRO-ASTRO consensus stating that oligometastatic disease (OMD) refers to cases with one to five metastatic lesions where all lesions can be safely treated. The definition is independent of the primary tumor type, the location of metastases and a history of disease-free survival [105]. Data on RCC have shown that OMD is a common presentation of metastatic RCC [106].

There are different types of OMD and they can be described according to the time of diagnosis of the primary and onset of metastases. A “synchronous OMD” is a case wherein the metastases and primary tumor are diagnosed simultaneously. “Metachronous OMD”, also termed “oligo-recurrence”, refers to patients found to have metastatic recurrence at least 3 months after the initial primary diagnosis. “Oligo-progression” (OPD) refers to patients with a history of responding or stable widespread metastatic disease but with the development of a limited number of progressing lesions. “Oligo-persistence” refers to cases of patients discovered to have a limited number of persistent lesions after systemic therapy, more commonly found in widely metastatic disease [105].

### 6.2. Metastasis-Directed Therapy and the Initial Studies on SBRT for Oligometastatic and Oligoprogressive Disease

The initial retrospective and observational studies on the use of metastasis-directed therapy (MDT) for patients with RCC OMD, which mainly involved surgical metastasectomy and SBRT, showed benefits. The results from metastasectomy papers revealed CSS and OS improvement [107,108,109], while studies on SBRT and OMD or OPD have shown durable LC rates of around 90% at 2 years, and the possibility of delaying the need to start or switch systemic therapy [110,111,112,113,114,115,116]. Lower doses, such as 25 Gy in 5–10 fractions, have been shown to result in relatively lower control at 55% 2-year in-field PFS [117].

Enthusiasm caused by the results of this retrospective research prompted the performance of prospective studies [118,119]. Most of the studies tried to focus on specific populations (e.g., OMD only, OPD only), although the inclusion criteria for each trial may differ slightly from the ESTRO-ASTRO consensus document.

### 6.3. Phase II Trials for Oligometastatic RCC

One of the first and more influential phase II trials was carried out by Tang et al. Patients had metachronous OMD and had no more than one previous systemic therapy. The trial allowed subsequent or longitudinal RT in cases of restricted progression (for ≤4 sites). SBRT and hypofractionated doses (if SBRT dosing was not feasible) were allowed in this study. At a median f/u of 17.5 months, 30 patients were enrolled. All had clear cell histology and underwent prior nephrectomy. Median PFS was 22.7 months and all patients completed radiation with unplanned breaks ≤7 days. A second subsequent course of radiation was given to 13 patients. The trial results were interpreted as supporting the strategy for prescribing longitudinal radiation as an alternative to systemic treatment [120]. Another trial advocating for a similar strategy was performed by Hannan et al. The study enrolled 16 metachronous and 7 synchronous OMD patients who were systemic therapy-naïve and had up to three extracranial sites. At 21.7 months median f/u, 91.3% of patients had >1-year freedom from systemic therapy. There were no grade 3–4 AEs but 1 patient had grade 5 toxicity of immune-related colitis [121].

A phase I/II trial by Siva et al. assessed the safety and efficacy of SBRT in OMD followed by eight cycles of Pembrolizumab in patients with clear cell histology. Up to 5 OMD sites and ≤2 lines of systemic therapy were allowed. Thirty patients were included. At 28 months median f/u, only four (13%) had grade 3 toxicity and no grade ≥4 toxicity. The 2-year freedom from local progression was 92%, the disease control rate was 83% and the objective response rate was 63%. The 2 yr OS and PFS were 74% and 45%, respectively [122]. Table 5 shows the published phase II trials while Table 6 shows the ongoing trials for RCC with OMD.

### 6.4. Phase II Trials for Oligoprogressive RCC

Cheung et al. assessed 37 patients with OPD on TKI therapy. Patients had received ≥3 months of TKI therapy and had ≤5 lesions. The median PFS was 9.3 months. The 1-year LC rate and systemic therapy rate of change was 93% and 47%, respectively [123]. Another phase II trial was performed by Hannan et al. They enrolled 20 patients who could have ≤3 OPD sites and were between their first and fourth lines of systemic treatment. Longitudinal SBRT was allowed if continually in an OPD state. At a median f/u of 10.4 months, the primary endpoint was reached with SBRT extending the ongoing systemic therapy by >6 months in 14 (70%) patients, above the pre-specified rate of 40%. Exploratory analysis showed 100% modified PFS for patients with ≤5 total metastases at time of SBRT vs. 50% for 50% for ≥6 metastases [124]. Table 7 shows the published phase II trials while Table 8 shows the ongoing trials for RCC with OPD.

### 6.5. Meta-Analyses, Reviews and Guidelines for OMD/OPD

A meta-analysis on SBRT was performed on patients with different types of OMD in 2019. Twenty-eight studies (all retrospective except one) were included. Cases involved both intracranial and extracranial lesions. Extracranial disease findings included a 59.7 cc median treatment volume, 1 yr LC of 89.1% and 1 yr survival rate of 86.8%, and the incidence of grade 3–4 AEs was only 0.7% [115]. A recent systemic review investigated different ablation studies. Fourteen of the eighteen studies focused on SBRT. The 1-year LC from four SBRT papers was 84.4% with a median LC of 87% for six SBRT studies and one cryotherapy study. The median OS for five SBRT and three IR ablation studies was 22.7 months. Five SBRT and two IR ablation papers revealed a median PFS of 9.3 months. Grade ≥3 toxicity occurred in only 1.7–10% [125].

Most patients in the trials had clear cell histology, favorable or intermediate IMDC risk scores [118] and one to three lesions treated. Due to this, some wonder about the applicability of SBRT in patients with non-clear cell histology, poor IMDC risk score and ≥4 sites of disease.

A consensus paper published in 2024 does provide expert opinion on patient selection and other management parameters in particular for OMD/OPD. An expert consensus (≥80% agreement) was reached on considering the number of metastases and time to progression as the main variables for choosing a specific treatment. Other areas with consensus were (1) not restricting SBRT to certain ages, histologies or locations; (2) using CT scans as the primary staging procedure; (3) using three lesions as the upper limit for those with OPD to undergo ablation; and (4) the concurrent prescription of ICIs with SBRT within 30 days. SBRT was also described as the treatment of choice for bone and adrenal metastases [126].

Similar to early-stage RCC, SBRT for oligometastatic sites has been mentioned or incorporated into some CPGs. The ESMO remarks that SBRT with metastasectomy and other ablative techniques are options for certain patients with low-burden metastatic disease after multidisciplinary team review. Its role is emerging in the synchronous OMD, metachronous OMD, OPD and mixed-response settings with immunotherapy [91]. The American Society of Clinical Oncology (ASCO) states that SBRT can be offered as part of MDT for patients with low-volume metastatic disease and to OPD patients to allow the continuation of immunotherapy [43]. The NCCN kidney cancer guide version 4.2024 cites SBRT, together with metastasectomy and other ablation techniques, as an option for both clear cell and non-clear cell cancers with oligometastatic disease, whether it is synchronous OMD, metachronous OMD or OPD [60].

### 6.6. SBRT for Oligometastatic Renal Cell Carcinoma Summary

In summary, the different trials and studies presented have shown that SBRT targeted to OMD and OPD sites has shown LC rates of >80%, a very low likelihood of moderate or severe toxicity at around 0–10% and a 1-year PFS of around 60–83%. SBRT may also delay the need for patients to start or switch systemic therapies [14,118,127]. As such, SBRT can be offered to non-surgical patients as a type of MDT. The further maturation of the aforementioned trials and the performance of other trials focused on specific populations or comparisons of different MDTs are awaited to further solidify SBRT in the treatment armamentarium of oligometastatic RCC.

The suggested SBRT dose regimens are the fractionations commonly used by the trials: 20–25 Gy/1, 36–39 Gy/3, 50 Gy/4 and ≥35–40 Gy/5. Alternative fractionation schedules, such as hypofractionation, were also introduced and can be used in cases where SBRT dose constraints can not be met [120,121,122,123,124]. A majority of experts recommend SBRT fractions to be given every other day [126].

## 7. Conclusions

Renal cell carcinoma was previously thought to be a radioresistant tumor, particularly with conventionally fractionated radiotherapy. Pre-clinical studies then showed improved responses using higher radiation doses per fraction. The development of SBRT subsequently made it possible to deliver this higher fractionation to patients with minimal risk.

For those with early-stage RCC, SBRT is a viable management option for non-operable cases. It provides excellent local control (>90%) and a good toxicity profile with minimal moderate-to-severe adverse events. SBRT is a commonly utilized MDT that is offered to patients with oligometastatic and oligoprogressive RCC. Treatment with SBRT may defer the need to initiate or replace systemic therapy, as it provides good control of the limited progressing disease. The probability of causing grade ≥3 adverse events is also low. The role of SBRT in both the early stage and oligometastatic stage may be further established with the maturation of concluded trials, and the completion of ongoing phase II or III trials. Also, more trials comparing different local treatments in early-stage disease and different MDTs in oligometastatic disease are needed. Another possible role for SBRT is in the cytoreductive setting for patients diagnosed with metastatic disease. It is postulated that SBRT of the kidney mass may lessen the tumor burden in the body and prevent further spread while also enhancing the immune response, particularly when given together with targeted therapy or immunotherapy. However, cytoreductive SBRT is still being investigated. Phase II trials are still ongoing or have only just recently finished accruing. The results are highly anticipated.

In conclusion, there is significant emerging data supporting the use of SBRT in RCC patients who are inoperable in the primary setting and those with oligometastatic or oligoprogressive disease as a metastasis-directed therapy. In the cytoreductive setting, though, trial results are needed before SBRT use can be supported. Future large-scale randomized trials are anticipated in order to further solidify the use of SBRT as a viable treatment option in both localized and metastatic RCC.

## Figures and Tables

**Table 1:** Alternative treatment options for inoperable cases of localized primary renal cell carcinoma.

Treatment	Possible Patient Selection Factors	Possible Limitations	Nuances
Active surveillance [4,58,59,60]	Common factors ^1^: - ≤4 cm (T1a) ^2^ [4,59,60,61] - significant comorbidities [61,62] - advanced age (e.g., >65–75 years old) [61,62,63]	- many patients may prefer treatment [17] - possible increased risk of death with surveillance vs. treatment shown in one database study [64]	- intervention considered once tumor size >4 cm or growth rate >0.5 cm per year or development of symptoms [59,60,65,66]
Interventional ablation	- <4 cm (T1a) - location is away from the renal hilum or proximal ureter [19]	- still has a small chance of periprocedural morbidity and anesthetic risk in high-risk patients due to the need for percutaneous or laparoscopic access [17]	- different techniques: cryotherapy [67,68] radiofrequency ablation (RFA) [67,69,70] microwave ablation [71]

^1^ Other factors have been mentioned in individual studies (e.g., radiologic appearance, histology) [72,73], but are not widely used. ^2^ Most studies and guidelines advocate for smaller tumors as excellent candidates for surveillance, but some have suggested that it could still be performed for select patients with tumors up to 7 cm [60,63].

**Table 2:** General considerations for SBRT in localized primary RCC.

Patient selection and evaluation:
- Suggested tumor size for SBRT: <5–8 cm [74], or ≤7 cm [17], but some protocols have included lesions up to 10 cm maximum size (T2a) [75] - Tumor not touching/invading bowel/stomach [17,75] - Consider as the next option if IR ablation is not feasible in medically inoperable patients [75] - Suggested pre-SBRT glomerular filtration rate (GFR) ≥30 mL/min [17,75,76] - Patients with solitary kidneys may undergo SBRT [17,76,77,78] - Nuclear medicine renal scan or renogram recommended prior to SBRT [17]

Simulation and Planning [17,74,75]:
- Stereotactic body frame or vac-lok bag, minimum half body - 4DCT (4-dimensional CT) scan with or without additional motion management - Intravenous contrast and MRI image registration when possible

Contouring [17,75,79]:
- Internal Target Volume (ITV) created from contouring and combining separate Gross Target Volumes (GTVs) on each respiratory phase of 4DCT simulation scans - Clinical Target Volume (CTV) expansion not usually recommended - Planning Target Volume (PTV) expansion: 5 mm (range 3–8 mm)

Radiation Doses [17,74,75,76,80]:
- Options: 25–26 Gy/1, 36–48 Gy/3 fractions, 35–50 Gy in 5 fractions - International Society of Stereotactic Radiosurgery (ISRS) fractionation: 26 Gy/1 fraction for <4–5 cm tumors and 42–48 Gy/3 or 40 Gy/5 for >4–5 cm tumors

Treatment [74,75]:
- Fractions generally given on non-consecutive days (about 40–48 h apart) with image guidance

Follow-up [17,75,76,79,81,82]:
- Every 3–4 months for the first year, 3–6 months for the second year and 3–12 months for a subsequent 3 years - Cross-axial imaging of the abdomen with kidneys and adrenals can be performed every 6 months. Renal function tests and surveillance scans involving the chest, at a minimum, can also be performed - Post-SBRT biopsy is generally not recommended, given insidious response to SBRT long-term

**Table 3:** Selected phase II trials investigating SBRT in localized primary RCC.

Author, Year (Trial Name)	Trial Schema	Results and Findings
Hannan, 2023 [74]	General inclusion/exclusion criteria: - biopsy-proven RCC - with radiographic growth - ≤5 cm mass Radiation Dose: - 36 Gy/3 or - 40 Gy/5 Patient Population: - 16 patients - median f/u of 3 years - median tumor size: 3.2 cm	Disease Control: - 95% LC at 1 year (primary endpoint) ^1^ - 100% LC by RECIST 1.1 at 1 year - 94% disease control rate at 3 years - no patient developed regional or distant metastases at 3 years Survival: - cancer-specific mortality was 0% at 3 years - OS at 3 years was 79% Toxicity: - no grade ≥2 acute or late toxicity - average GFR declined (65.6 to 55.4 mL/min at 1 yr) Other findings: - tumor viability decreased (4.6% to 0.7% at 1 yr) - spatial protein and gene expression analyses revealed that radiation induced cellular senescence
Siva, 2024 (Fastrack II) [75]	General inclusion/exclusion criteria: - biopsy-proven RCC - ≤10 cm tumors - not in contact with bowel - not suitable for thermal ablation - pre-SBRT GFR ≥30 mL/min Radiation Dose: - ≤4 cm tumors: 26 Gy/1 - 4–10 cm: 42 Gy/3 Patient Population: - 70 patients - median f/up of 43 months - median tumor size: 4.6 cm	Disease control: - 100% LC at 1 year by RECIST 1.1 (primary endpoint) - freedom from distant failure was 97% and 82% at 1 and 3 years Survival: - cancer-specific survival was 100% - OS was 99% at 1 year and 82% at 3 years Toxicity: - seven patients (10%) with grade 3 toxicity (nausea, vomiting, abdominal, flank or tumor pain) - no grade ≥4 AEs - average eGFR reduction of 8.4 mL/min at 1 year - decline in renal function plateaued after 2 years
Swaminath, 2023 (RADSTER—abstract only) [80]	Population: - biopsy-proven RCC - ≤4 cm tumors Treatment Dose: - RFA: percutaneous with two cycles up to 8 min each - SBRT: 25 Gy (25 Gy/1) Patient Population: - 24 patients - three RFA patients crossed over to SBRT (due to technical inability)	Disease Control: - no radiographic (RECIST) local failure in 1 year for both groups - RFA tumors, more likely lose arterial enhancement - post-treatment biopsies at 1 year: RFA had higher rates of complete pathologic response (7/7) compared to SBRT (4/13) Survival: - no patients developed distant disease or died from RCC Toxicity: - only one grade 2 acute pain flare (SBRT patient) - no late toxicity in 1 year for both groups - no difference in mean eGFR reduction (RFA: −3 mL/min; SBRT: −5.3 mL/min; *p* = 0.07)

^1^ local control defined by reduction in tumor growth rate and pathology. f/u—follow-up; GFR—glomerular filtration rate; LC—local control; OS—overall survival; RFA—radiofrequency ablation; RECIST—response evaluation criteria in solid tumors; RCC—renal cell carcinoma; SBRT—stereotactic body radiotherapy.

**Table 4:** Phase II trials investigating cytoreductive SBRT.

Trial Name	Radiation Dose	Timing of RT	Systemic Therapy	Primary Objective
Cytoshrink NCT04090710	30–40 Gy/5	SBRT in between first and second cycles	Ipilimumab + Nivolumab	- PFS
Samurai ^1^ NCT05327686	42 Gy/3	SBRT prior to cycle 1	(1) Ipilimumab + Nivolumab (2) Pembrolizumab + Axitinib (3) Avelumab + Axitinib (4) Pembrolizumab + Lenvatinib	- Nephrectomy and radiographic PFS

^1^ includes node-positive unresectable cases. PFS—progression-free survival; RT—radiotherapy; SBRT—stereotactic body radiotherapy.

**Table 5:** Selected trials investigating SBRT for oligometastatic disease.

Author, Year (Trial Name—Phase)	Trial Schema	Patient Population	Endpoints and Toxicities
Tang, 2021 (phase II) [120]	General inclusion/exclusion criteria: - ≤5 sites - ≤1 line of systemic therapy - sequential SBRT allowed if still OMD (≤4 sites) Radiation Doses: - SBRT of <5 fractions with >7 Gy per fraction (most common 50 Gy/4) - 60–70 Gy/10 - 52–67.5 Gy/15	- 30 metachronous OMD patients - median f/u 17.5 months - all were RCC and underwent nephrectomy - 29 (97%) had favorable or intermediate IMDC risk - 28 (94%) had 1–2 sites treated on first round of SBRT - 13 (43%) patients had a second RT course (all had 1–2 sites treated)	Endpoints: - all patients completed the first round of RT with ≤7 days unplanned breaks (PE) - median PFS 22.7 months (PE) - 1-year PFS of 64% - 1-year OS of 100% - 1-year freedom from new lesions of 67% - 1 year systemic therapy-free survival of 82% - 1-year LC of 97% Toxicity: - three (10%) had toxicity - two had grade 3 AEs of back pain and muscle weakness - one had a grade 4 AE (hyperglycemia)
Hannan, 2022 (phase II) [121]	General inclusion/exclusion criteria: - ≤3 sites - systemic therapy-naïve - favorable or intermediate IMDC risk - sequential SBRT allowed if still OMD Radiation Doses: - 20–25 Gy/1 - 36–39 Gy/3 - 35–40 Gy/5	- 23 patients (70% metachronous; 30% synchronous) - median f/u of 21.7 months - 22 (95.7%) underwent nephrectomy - 19 (82.6%) clear cell RCC - 21 (91.3%) had 1–2 OMD sites treated with SBRT	Endpoints: - freedom from systemic therapy for >1 year of 91.3% (PE) - 1-year PFS of 82.6% - 1-year LC of 100% - 1-year CSS and 95.7% - 1-year OS of 95.7% Toxicity: - one grade 2 AE - no grade 3–4 AEs - one grade 5 AE (immune-related colitis)
Siva, 2022 (RAPPORT–phase I/II) [122]	General inclusion/exclusion criteria: - ≤5 sites - at least one lesion SBRT-deliverable - ≤2 lines of systemic therapy Radiation Doses: - 20 Gy/1 (77% of patients) - 30 Gy/10 Systemic therapy: - eight cycles of Pembrolizumab every 3 weeks after SBRT	- 30 patients - median f/u of 28 months - all had favorable or intermediate IMDC risk - 23 (77%) were systemic treatment-naive - median of three OMD sites treated per patient	Endpoints: - 2-year freedom from local progression of 92% - disease control rate of 83% - objective response rate of 63% - estimated 1- and 2 yr OS of 90% and 74% - 1- and 2 yr PFS of 60% and 45% Toxicity (PE): - four (13%) grade 3 AEs (pneumonitis, dyspnea, high ALK/ALT) - no grade 4–5 AEs

AE—adverse event; ALT—alanine transaminase; ALK—alkaline phosphatase; CSS—cancer-specific survival; f/u—follow-up; IMDC—International Metastatic RCC Database Consortium; LC—local control; PE—primary endpoint; PFS—progression-free survival; OMD—oligometastatic disease; OS—overall survival; SBRT—stereotactic body radiotherapy.

**Table 6:** Select ongoing trials investigating SBRT for oligometastatic disease.

Sponsor (Trial Name)—Phase	Trial Schema	Metastatic Sites and Systemic Therapy	Primary Objective	Date of Primary Completion
ECOG-ACRIN (SOAR trial)—phase III (NCT05863351)	Systemic therapy vs. SBRT until progression then systemic therapy	- two to five sites - standard systemic (investigator discretion)	- OS - toxicity incidence	December 2030 (estimated)
MDACC (ASTROs trial)—phase II (NCT06004336)	SBRT vs. SBRT followed by systemic therapy	- ≤5 sites - Pembrolizumab for 17 cycles	- PFS	January 2029 (estimated)
Consorzio Oncotech—phase II (NCT05578664)	MDT (surgery or RT) vs. Pembrolizumab + MDT (surgery or RT)	- ≤3 sites - Pembrolizumab for 9 cycles	- RFS	October 2024 (estimated)
University of Chicago—pilot (NCT02542202)	SBRT to synchronous or metachronous OMD	- ≤5 sites - none	- grade ≥4 AEs	March 2023 (actual)

AE—adverse event; ECOG-ACRIN – Eastern Cooperative Oncology Group (ECOG-ACRIN Cancer Research Group; OMD—oligometastatic disease; OS—overall survival; PFS—progression free survival; RFS—recurrence free survival; SBRT—stereotactic body radiotherapy; MDACC – MD Anderson Cancer Center; MDT—metastasis-directed therapy.

**Table 7:** Select SBRT trials on oligoprogressive disease.

Author, Year—Phase	Trial Schema	Patient Population	Results and Findings
Cheung, 2021—phase II [123]	General inclusion/ exclusion criteria: - ≤5 sites - ≥3 mos on TKI - clear cell - favorable or intermediate IMDC risk Radiation Doses: - 40 Gy/5 (median BED10 72 Gy)	- 37 patients - 35 patients were on Sunitinib - 34 (92%) had 1–2 sites	**Endpoints:** - 1-year incidence of changing systemic therapy of 47% - median PFS 9.3 mos - median time to change in systemic therapy of 12.6 mos - 1-year LC rate of 93% (PE) **Toxicity:** - no grade 3–5 toxicities
Hannan, 2022—phase II [124]	General inclusion/exclusion criteria: - ≤3 sites (≤30% of all sites) - on first-to-fourth line systemic therapy - favorable or intermediate IMDC risk - allowed subsequent SBRT if still OPD Radiation Doses: - ≥25 Gy/1 - ≥36 Gy/3 - ≥40 Gy/5	- 20 patients - median f/u of 10.4 months - all had only 1–2 sites treated with SBRT initially	**Endpoints:** - SBRT extended current systemic therapy by >6 mos in 14 (70%) patients (PE) - SBRT-aided systemic therapy median PFS was 24.4 mos - median time from SBRT to start of new systemic therapy or death was 11.1 mos - LC rate of 100% at median follow-up - median survival not reached **Toxicity:** - one (5%) had grade 3 toxicity - no grade 4–5 toxicity

f/u—follow-up; IMDC—International Metastatic RCC Database Consortium; LC—local control; PE—primary endpoint; PFS—progression-free survival; OPD—oligoprogressive disease; SBRT—stereotactic body radiotherapy; TKI—tyrosine kinase inhibitor.

**Table 8:** Select ongoing trials investigating SBRT for oligoprogressive disease.

Sponsor (Trial Name)—Phase	Trial Schema	Systemic Therapy	Primary Objective	Date of Primary Completion
Yale University—phase II (NCT-04974671)	- SBRT to patients on ICI-regimen (within 3 months) - other local therapy allowed - SBRT to at least one lesion	- ICI	- PFS	October 2025 (estimated)
Centre Francois Baclesse (GETUG-STORM-01)—phase II (NCT-04299646)	systemic therapy vs. SBRT + Systemic therapy	- TKI or mTOR inhibitor - immunotherapy	- PFS	September 2025 (estimated)

ICI—immune checkpoint inhibitor; mTor—mammalian target of rapamycin inhibitors; PFS—progression-free survival; SBRT—stereotactic body radiotherapy; TKI—tyrosine kinase inhibitor.

## Data Availability

No new data were created or analyzed in this study. Data sharing is not applicable to this article.
